# Multi-modal MRI of hippocampal morphometry and connectivity after pediatric severe TBI

**DOI:** 10.1007/s11682-023-00818-x

**Published:** 2023-11-13

**Authors:** Jose M. Guerrero-Gonzalez, Gregory R. Kirk, Rasmus Birn, Erin D. Bigler, Katherine Bowen, Aimee T. Broman, Bedda L. Rosario, Warwick Butt, Sue R. Beers, Michael J. Bell, Andrew L. Alexander, Peter A. Ferrazzano, ADAPT MRI Biomarkers Investigators, Ranjit Chima, Robert Clark, Nikki Ferguson, Mary Hilfiker, Kerri LaRovere, Iain Macintosh, Darryl Miles, Kevin Morris, Nicole O’Brien, Jose Pineda, Courtney Robertson, Karen Walson, Nico West, Anthony Willyerd, Jerry Zimmerman, Brandon Zielinski

**Affiliations:** 1 Department of Medical Physics, University of Wisconsin School of Medicine and Public Health, Madison, WI USA; 2 Department of Psychiatry, University of Wisconsin School of Medicine and Public Health, Madison, WI USA; 3 Department of Psychology and Neuroscience Center, Brigham Young University, Provo, UT USA; 4 Seattle Children’s Hospital, Seattle, WA USA; 5 Department of Biostatistics, University of Wisconsin–Madison, Madison, WI USA; 6 Department of Epidemiology, School of Medicine, University of Pittsburgh, Pittsburgh, Pennsylvania USA; 7 Department of Critical Care, Faculty of Medicine, Melbourne University, Melbourne, Australia; 8 Department of Psychiatry, School of Medicine, University of Pittsburgh, Pittsburgh, Pennsylvania USA; 9 Department of Pediatrics, Children’s National Medical Center, Washington, DC USA; 10 Department of Pediatrics, University of Wisconsin School of Medicine and Public Health, Madison, WI USA; 11 Waisman Center, University of Wisconsin–Madison, 1500 Highland Avenue, Madison, WI 53705 USA; 12 Department of Neurology & Department of Psychiatry, University of Utah, Salt Lake City, UT USA

**Keywords:** Hippocampus, TBI, Connectivity, Fornix, Memory

## Abstract

This investigation explores memory performance using the California Verbal Learning Test in relation to morphometric and connectivity measures of the memory network in severe traumatic brain injury. Twenty-two adolescents with severe traumatic brain injury were recruited for multimodal MRI scanning 1–2 years post-injury at 13 participating sites. Analyses included hippocampal volume derived from anatomical T1-weighted imaging, fornix white matter microstructure from diffusion tensor imaging, and hippocampal resting-state functional magnetic resonance imaging connectivity as well as diffusion-based structural connectivity. A typically developing control cohort of forty-nine age-matched children also underwent scanning and neurocognitive assessment. Results showed hippocampus volume was decreased in traumatic brain injury with respect to controls. Further, hippocampal volume loss was associated with worse performance on memory and learning in traumatic brain injury subjects. Similarly, hippocampal fornix fractional anisotropy was reduced in traumatic brain injury with respect to controls, while decreased fractional anisotropy in the hippocampal fornix also was associated with worse performance on memory and learning in traumatic brain injury subjects. Additionally, reduced structural connectivity of left hippocampus to thalamus and calcarine sulcus was associated with memory and learning in traumatic brain injury subjects. Functional connectivity in the left hippocampal network was also associated with memory and learning in traumatic brain injury subjects. These regional findings from a multi-modal neuroimaging approach should not only be useful for gaining valuable insight into traumatic brain injury induced memory and learning disfunction, but may also be informative for monitoring injury progression, recovery, and for developing rehabilitation as well as therapy strategies.

**Supplementary information:**

The online version contains supplementary material available at 10.1007/s11682-023-00818-x.

## Introduction

Impaired memory function following traumatic brain injury (TBI) has long been known as the most common, persisting cognitive deficit (Schacter & Crovitz, 1977). The hippocampal formation is the centerpiece for learning and memory with its extensive afferent and efferent connections with the rest of the brain, but its medial temporal lobe location makes it particularly vulnerable to traumatic injury and TBI-induced memory impairment (C. N. Smith et al., 2013). From a quantitative neuroimaging perspective, the volume of the hippocampus as a marker of hippocampal damage has been consistently related to various memory deficits, especially in severe-TBI. The relationship is however not exceptionally robust (Bigler et al., 1997; Himanen et al., 2005; Tomaiuolo et al., 2004).

There are several possible explanations why assessing the size of the hippocampus as a predictor of memory outcome following TBI falls short of being a robust indicator of the level of memory impairment. The memory network is complex including not only the hippocampus, but fornix, mammillary bodies, anterior thalamic projections and aspects of the cingulum bundle (Budson & Price, 2005). Furthermore, the hippocampus is a structure that interfaces with all primary sensory and motor cortical regions as well as association cortices and therefore, poses a complex neural structure throughout the brain (Dalton et al., 2022; Ekstrom & Ranganath, 2018; Lavenex & Amaral, 2000; Maller et al., 2019; Raut et al., 2020).

In the past decade, with advanced methods for image analysis, it has become important to not only examine where a specific TBI-induced lesion or focal abnormality may be identified, but also how neural networks are damaged and where in the network may be most affected (Gordon et al., 2018; Rangaprakash et al., 2018; Yan et al., 2016). In terms of traumatically-induced hippocampal pathology, a multimodality neuroimaging approach is most appropriate as it takes into account not only the target structure, but also the neural network associated with the hippocampus (Irimia et al., 2017). As such, in the current study we employed volumetric as well as structural and functional neuroimaging analyses that examined the memory network in relation to neuropsychological learning and memory performance on the California Verbal Learning Test (CVLT-C/II) in children recovering from severe traumatic brain injury and typically developing controls.

## Methods

### Participants

Children 11–18 years old and enrolled in the Approaches and Decisions in Acute Pediatric TBI (ADAPT) trial were recruited for inclusion in this study. ADAPT enrolled 1000 severe TBI children (post-resuscitation Glasgow Coma Scale (GCS) < 8 (Bell et al., 2022; Ferrazzano et al., 2019; Kochanek et al., 2022)). Typically developing (TD) participants without history of TBI or neuropsychiatric diagnoses were recruited at the University of Wisconsin – Madison (UW). The TBI group consisted of 22 patients (12 females) between the ages of 11.6 and 18.9 years (mean ± SEM, 15.7 ± 2.1 years) at time of MRI scanning, recruited from thirteen sites. These were subjects that met the inclusion criteria from a subset of ADAPT participating sites and agreed to follow up assessments. The mechanism of injury was predominantly motor vehicle accident (77%) with the remainder due to falls and other accidental injuries. The TD group had 49 subjects (25 females) between the ages of 9.0 and 18.0 years (mean 13.45 ± 2.8 years).

### California verbal learning test

The California Verbal Learning Test is a standardized list learning task, for both children—Children’s Edition (CVLT-C; (Delis et al., 1994)) and those 16 and older – Second Edition (CVLT-II; (Delis et al., 2000)). Since the ADAPT cohort examined in this investigation spanned children 11 to 18 years of age, those under 16 received the CVLT-C and those 16 and older, received the CVLT-II. While the word lists are different, the format is similar and only the total recall T-score from trials 1–5, based on the normative data based on the child’s age was used as a memory index. The CVLT-C/CVLT-II is a widely used and well-validated test of learning and memory that involves learning a standardized list of words read to examinees over several trials, followed by several conditions of recall and recognition. The total recall during trials 1–5 (“CVLT T-score”), chosen as our variable of interest, is considered a measure of verbal learning and immediate memory. Eighteen TBI subjects completed CVLT testing and were included in this analysis.

### Brain imaging

Prior to subject enrollment, sites were provided scanner-specific protocols to be implemented on their system. Protocols were harmonized to conform to those similar in the multi-site Transforming Research and Clinical Knowledge in TBI (TRACK-TBI) study. Scanning procedures were disseminated to all participating sites. Sites were required to collect phantom data using the provided protocol and send to UW to verify compliance. Once approved, sites enrolled adolescent TBI participants. Imaging was performed 12–25.5 months post injury with a mean interval between injury and MRI scanning of 20 ± 4.44 months. Data from T1-weighted images, diffusion tensor imaging (DTI), and resting-state functional MRI (rs-fMRI) were used in this analysis. Based on visual quality inspection, three participants had unusable diffusion and rs-fMRI data due to severe susceptibility distortion artifacts from what appeared to be wearing of braces. We assessed and reported (Guerrero-Gonzalez et al., 2022) cross site variability in diffusion measurements using the NIST PVP diffusion  (Boss et al., 2015) phantom at 6 sites which demonstrated consistency across sites. Given the stability in these metrics and the small sample size, site was not included as covariate in subsequent analyses. Relevant vendor-specific scanning parameters are shown in Table 1.

**Table 1:** Vendor specific scanning settings for T1-weighted, DTI, and fMRI scans, conforming to TRACK-TBI study

GE				Siemens				Philips			
Parameter	T1	DTI	fMRI	Parameter	T1	DTI	fMRI	Parameter	T1	DTI	fMRI
Sequence Name	BRAVO	DTI	rs_fMRI	Sequence Name	MP-RAGE	DTI	rs_fMRI	Sequence Name	MP-RAGE	DTI	RS fMRI
Orientation	Sagittal	Sagittal	Sagittal	Orientation	Sagittal	Sagittal	Sagittal	Orientation	Sagittal	Sagittal	Sagittal
FOV	256	240	220	FOV	256	240	220	FOV FH (mm)	256	240	220
Freq	256	96	64	Matrix	256 × 256	96 × 96	64 × 64	Matrix scan	256 × 256	96 × 96	64 × 64
Phase	256	96	64								
Voxel Size—Freq	1	2.5	3.4	Voxel Size—Freq	1	2.5	3.4	Voxel Size—Freq	1	2.5	3.4
Voxel Size—Phase	1	2.5	3.4	Voxel Size—Phase	1	2.5	3.4	Voxel Size—Phase	1	2.5	3.4
Slice Thickness	1	2.5	4	Slice Thickness	1	2.5	4	Slice Thickness	1	2.5	4
# slices	192	60	42	Slices	192	64	40	# Slices	160	64	42
Phase Encoding	A/P	A/P	A/P	Phase Direction	A > > P	A > > P	A > > P	Fold- over direction	AP	AP	AP
TR	Min (8.2)	8500	2000	TR	2000	9000	2000	TR	shortest	8500	2000
TE	Min (3.2)	Min (82.1)	25	TE	2.56	86	25	TE	shortest	103	25
b-value	NA	1300	NA	b-value	NA	1300	NA	Max **b-factor / b-value**	NA	1300	NA
# **Diffusion directions**	NA	64	NA	# **Diffusion directions**	NA	64	NA	# **Diffusion directions**	NA	64	NA
Slice **per Location**	NA	NA	154	Measurements	NA	1	154	# Dynamic Scans	NA	NA	154

### Memory network morphometry and connectivity

The multi-modal framework detailing several pre- and post-processing steps, and specific commands used is presented in the Supplement. Major relevant steps of the multimodal approach are provided next.

Hippocampal volume was derived from FreeSurfer (Fischl et al., 2002) segmentations of the T1-weighted images (*recon-all* command). Mean diffusion tensor fractional anisotropy (FA) was sampled (MRtrix’s *tcksample* command) from TractSeg (Wasserthal et al., 2018) fornix segmentations (*TractSeg* command) of pre-processed DTI scans. At this stage, one TBI participant was excluded due to a large lesion in region of the fornix precluding accurate segmentation. Another TBI subject was excluded due to visually-assessed excessive motion artifact on the DTI scan.

**Fig. 1:**
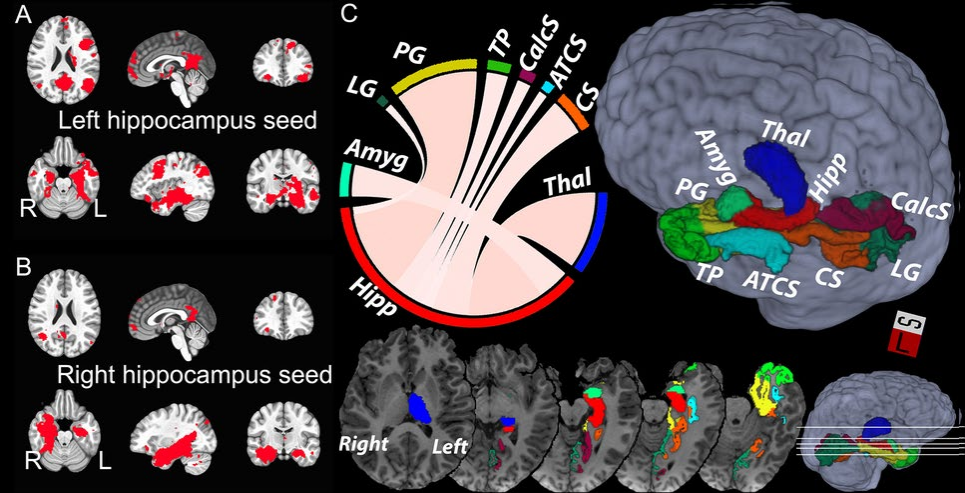
Functional and structural hippocampal networks in TD group. **A**,**B**: Network of brain regions with significant functional connectivity to the hippocampus. **C**: Left hippocampal structural connectome (6 of the same regions were found for the right hippocampus). Abbreviations: parahippocampal gyrus (PG, note, the FreeSurfer definition of PG includes also the entorhinal cortex), thalamus (Thal), the medial occipito-temporal and lingual sulcus (collateral sulcus, CS), amygdala (Amyg), temporal pole (TP), calcarine sulcus (CalcS), anterior transverse collateral sulcus (ATCS), lingual part of the medial occipito-temporal gyrus (lingual gyrus, LG)

Hippocampus functional connectivity (FC) was produced using the AFNI software package from averaged pre-processed fMRI data over hippocampus masks, followed by computing the temporal Pearson’s correlation with all other voxel time series. The network of brain regions significantly connected to each hippocampus (“hippocampal network”, Fig. 1) was determined by computing a one-sample t-test of Fisher-Z transformed voxel-wise connectivity maps in the control subjects. Voxels where the group-level FC exceeded Bonferroni corrected p-value of 0.05 were considered significantly connected. The hippocampal FC in each TBI subject was then averaged over each hemisphere’s hippocampal network.

A hippocampus-specific structural connectome was derived by first producing individual connectomes using spherical-deconvolution informed filtering of tractograms-2 (SIFT2) for whole-brain probabilistic fiber tracking (Smith et al., 2015; Tournier et al., 2019) combined with 164 gray matter regions from the Destrieux atlas (Destrieux et al., 2010; Fischl et al., 2002). The measure of structural connectivity was the fiber bundle capacity (FBC), which represents the ability of a white matter pathway to carry information (Smith et al., 2020). Full-brain structural connectomes were averaged across participants in the TD group. Subsequently, hippocampus-specific connections were extracted from the mean connectome. This connectome was sorted in descending order according to FBC. Finally, the top 5% connections were selected to form the hippocampus connectome. This definition was used to study connectivity in the TBI cohort.

Group comparisons of neuroimaging measures (hippocampus volume, fornix FA) and associations with CVLT (hippocampus volume, fornix FA, FBC, functional connectivity) were tested using linear regression. Six out of the 8 regions representing the top 5% in the left hippocampus connectome (Fig. 1) also appear on the right. The lingual and medial gyri and the anterior transverse collateral sulci were found only in the left hippocampal connectome, while the putamen and the pallidum were found only in the right. See Figure S3 (Supplement) for all regions included in the right-side hippocampal connectome. Note, the FreeSurfer definition of the parahipocampal gyrus includes also the entorhinal cortex and the thalamus encompasses the whole of the thalamus proper region.

## Results

### Hippocampal volume

**Fig. 2:**
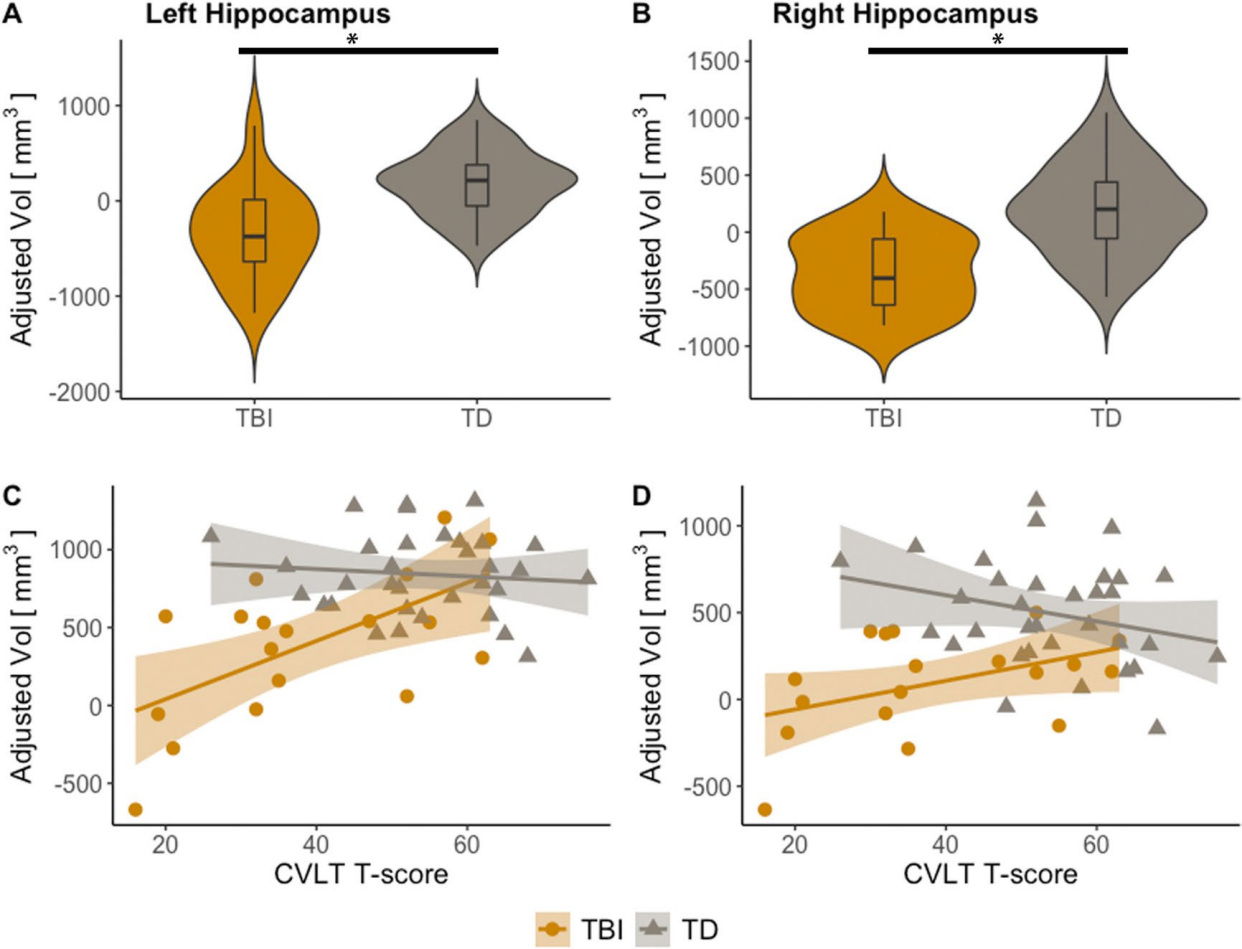
Hippocampus volume by group and memory performance score. **A**-**B**, group comparison of left and right hippocampus volume between TBI (yellow) and control cohort (gray), adjusted for age and sex; *p < 0.05. **C**-**D**, left and right hippocampus volume as a function of CVLT T-score for TBI (yellow) and control cohorts (gray), adjusted for age and sex

Volume group comparisons are shown in Fig. 2. Group comparisons included twenty-two TBI participants. Adjusted for age, sex, brain and intracranial volumes, hippocampal volume was smaller in TBI (left-hippocampus: se = 1.080 × 10^2^, t = 4.516, p < 0.001; right-hippocampus: se = 8.997, t = 5.126, p < 0.001). Figure 2 also shows partial residual plots for CVLT. This part of the analysis includes all eighteen TBI participants that completed CVLT testing. Adjusted values reflect CVLT-fitted value plus the residual. Positive significant associations were found in TBI (left-hippocampus: se = 4.983, t = 4.065, p < 0.001, R^2^ = 0.3732; right-hippocampus: se = 4.525, t = 2.188, p = 0.03, R^2^ = 0.3488). Associations for TD participants were non-significant. For reference, a group comparison revealed a significantly lower CVLT score in the TBI group (TBI mean: 38.4, TD mean: 53.5; Welch Two Sample t-test: t = -4.0352, df = 30.836, p-value = 0.0003337).

### Fornix FA

**Fig. 3:**
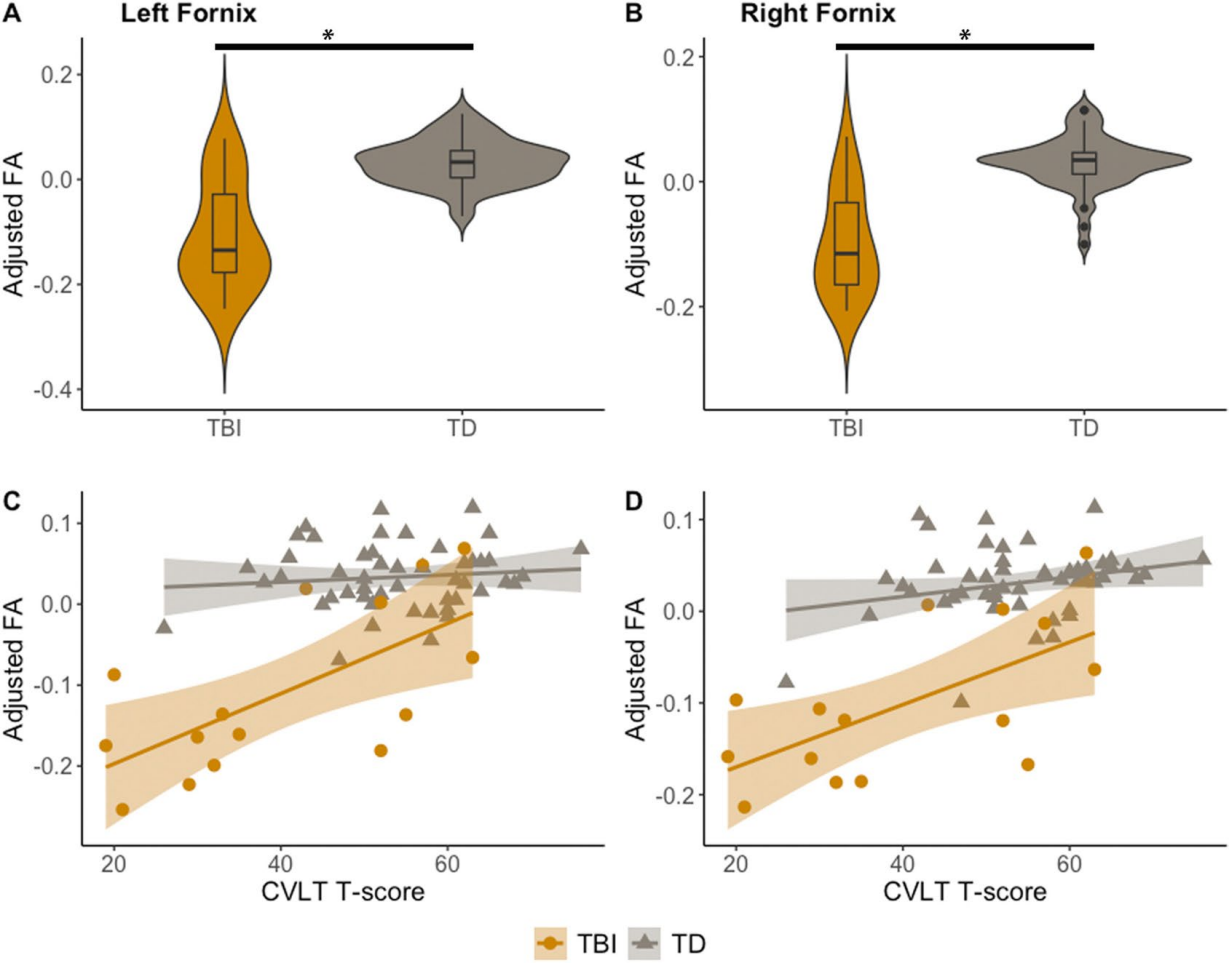
Fornix FA by group and memory performance. **A**-**B**, group comparison of left and right fornix FA between TBI (yellow) and control cohort (gray), adjusted for age and sex; *p < 0.05. **C**-**D**, left and right fornix FA as a function of CVLT T-score for TBI (blue) and control cohorts (gray), adjusted for age and sex

Fornix FA group comparisons are shown in Fig. 3 (age- and sex-adjusted). Group comparisons included seventeen TBI participants. Adjusted-FA was significantly smaller in TBI (left-hippocampus: se = 0.01871, t = -7.121, p < 0.001; right-hippocampus: se = 0.016198, t = -7.398, p < 0.001). Out of the seventeen TBI participants in the group analysis, fifteen had completed CVLT testing. For these participants, Fig. 3 also shows partial residual plots and model fits for CVLT. Adjusted values reflect CVLT-fitted value plus the residual. Significant positive associations were found in TBI (left-hippocampus: se = 0.001520, t = 2.845, p = 0.0159, R^2^ = 0.3275; right-hippocampus: se = 0.001230, t = 2.662, p = 0.0221, R^2^ = 0.3642). Associations for TD participants were non-significant.

### Hippocampal connectivity

**Fig. 4:**
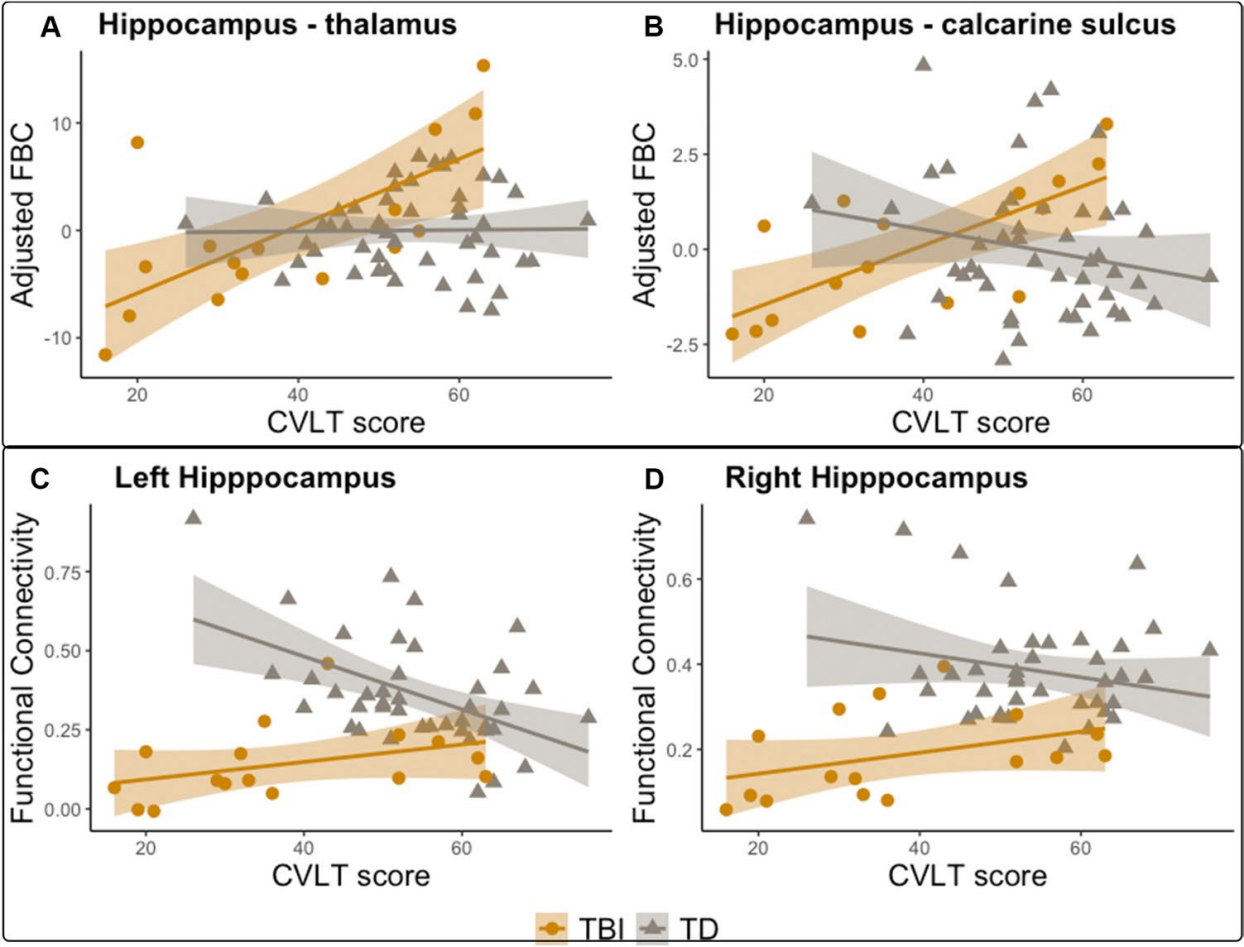
Hippocampal connectivity and CVLT. Top panel: Structural connectivity and memory performance. Fiber bundle capacity as a function of CVLT T-score for TBI (yellow) and control cohort (gray), adjusted for age and sex, for hippocampus with (**A**) thalamus and with (**B**) calcarine sulcus. Bottom panel: Association between hippocampus functional network connectivity and memory performance for left (**C**) and right (**D**) hippocampi. Spearman rank correlation between Hippocampal FC and CVLT T-score is shown for TBI (yellow) and control (gray) subjects

After correcting for age and sex, significant associations were found for CVLT in TBI with left-hippocampus FBC to ipsilateral calcarine sulcus (R^2^ = 0.4333) and thalamus (R^2^:0.3625) (Fig. 4). These associations were statistically significant to p < 0.05 after FDR multiple-comparisons correction. Also, average hippocampus FC in TBI was significantly associated with CVLT (Fig. 4) in the left hemisphere (p = 0.039, R^2^ = 0.027), and associated at a trend-level in the right hemisphere (p = 0.057, R^2^ = 0.023). A significant correlation was found for TD participants in the left hippocampus (p < 0.001, R^2^ = 0.25). These analyses included sixteen participants that had diffusion and rsfMRI imaging data and also had completed CVLT assessments.

## Discussion

This work employed a multi-modality MRI approach to study the memory network in relation to neuropsychological memory and learning performance on the CVLT following severe TBI.

### Hippocampal volumetrics after TBI

We found significant reductions in hippocampal volume in TBI subjects compared to controls, and in TBI subjects the hippocampal volume was associated with memory and learning performance on the CVLT. The hippocampus has been consistently identified as a brain structure affected by TBI, and the extent of hippocampal atrophy has been associated with severity of injury (Beauchamp et al., 2011; King et al., 2019; Tasker et al., 2005; Tate & Bigler, 2000). Importantly, our analysis adjusted for brain volume thus reflects hippocampal-specific atrophy. Biomechanically, the position of the hippocampus within the medial temporal lobe in association with the boney structures of the middle cranial fossa and the inferior horn of the lateral ventricular system, make it especially vulnerable to shear-strain-deformation injury from TBI (Zhou et al., 2022). Selective hippocampal atrophy was similarly found by (Wilde et al., 2007) in their study of 16 children with moderate-to-severe TBI. Given its central role in memory formation and retrieval, hippocampal volume loss has been investigated as a marker of TBI-induced memory dysfunction, though most prior studies have been performed in adults, so information on children with severe-TBI is limited. In a study of 86 subjects age 16–65 years with any-severity TBI, (Tate & Bigler, 2000) found significant reductions in hippocampal volume in TBI compared to controls 2 months post-injury. The degree of atrophy in these regions correlated with injury severity and the General Memory Index of the Wechsler Memory-Scale. Similarly, a study of 129 children age 8–15 years with any-severity TBI (15 severe-TBI subjects), (DeMaster et al., 2017) found atrophy in hippocampal subregions was associated with TBI injury severity and memory performance 6 weeks post-injury. Adding to these studies performed early after injury, our findings suggest that the association between hippocampal atrophy and memory dysfunction is sustained up to 1–2 years post-injury in children with severe TBI.

### Alterations in fornix microstructure

FA in the fornix of the TBI group significantly decreased compared to the TD group. In the TBI group, but not in the TD cohort, reduced fornix FA was associated with memory and learning impairment on the CVLT. Being the major carrier of efferent projections from the hippocampus and connecting it to structures such as mammillary bodies, thalamus, and temporal lobe, the fornix plays an important role in the hippocampal network and in memory function (Bigler et al., 2010). The fine fornix tracts straddling both cerebral hemispheres are especially vulnerable to physical shearing forces (Tate & Bigler, 2000), and TBI has long been associated with fornix atrophy (Gale et al., 1993). Decreased fornix FA at the chronic stage of TBI may reflect disruptions in tissue microstructure such as loss of axonal fiber coherence or density as well as demyelination. To investigate this, in a secondary analysis we assessed fornix-specific apparent fiber density (AFD) voxel maps, using the *afdconnectivity* tool within MRtrix (Tournier et al., 2019) with the ‘*-afd_map’* option. This uses the input fornix streamlines to identify the fixels belonging to the tract-of-interest, computes the fixel-specific AFD as the fiber orientation distribution (fODF) lobe integral and outputs it as a 3D scalar map. As described in this publication (Smith et al., 2020), integrating a discrete fODF lobe is proportional to the MR-visible intra-cellular tissue volume at high b-values in the direction of that lobe. We compared fornix AFD between the TBI and TD groups and found a significant reduction in AFD for the TBI group. See Figure S5 (Supplement). Mechanisms such as loss of axonal fiber coherence or density may be related to direct injury in the fornix or atrophy nodes connected by it like the hippocampus or mammillary bodies. Our findings of decreased fornix FA associated with worse performance on CVLT in TBI are consistent with a study of DTI that found abnormal FA in several regions of the brain in adult TBI patients, but only fornix FA correlated with performance in associative memory and learning tasks (Kinnunen et al., 2011). In another study of diffuse TBI, adult patients were observed to have globally decreased FA in the brain, but regional analyses revealed that lower fornix FA was associated with poorer declarative memory (Palacios et al., 2011). More generally, (Adnan et al., 2013) suggested a critical role for fornix integrity in the development of memory impairments after moderated-to-severe TBI in adults. Their findings consistently showed lower fornix FA accompanied memory deficits compared to healthy controls. These numerous findings including our own linking decreased FA in the fornix to declining memory function in TBI are indicative that fornix FA may provide a robust imaging marker for TBI-induced memory deficits.

### Hippocampal network connectivity

Statistically significant linear associations were found for memory and learning with respect to connectivity strength of hippocampus to the thalamus and the calcarine sulcus. These were found for the left hemisphere only, and the majority of the subjects in our study were right-handed. CVLT, as its name implies is an auditorily presented, verbal learning and memory task. The *material-specificity* theory posits that left and right hippocampus differentially process verbal and visual memory, supported by many studies, including elegant studies of temporal lobe epilepsy in children which demonstrate reductions specifically in verbal memory after temporal lobe resections of the left hippocampus (Law et al., 2017; Sepeta et al., 2018), and broader functional imaging studies of medial temporal lobe and hippocampus (Dalton et al., 2016; Golby, 2001). CVLT is therefore dependent on the integrity of left hemisphere language and memory networks (Lezak, 2012), and would be expected to relate to left hemisphere hippocampal network analyses as we found in the current investigation of children with severe-TBI. Interestingly, hippocampal connectivity with the calcarine sulcus, a visual processing region, was significantly associated with verbal memory performance in our study. These findings are consistent with those from recent investigations of hippocampal structural connectivity that report strong patterns of connectivity between the hippocampus and these visual cortex regions (Dalton et al., 2022; Maller et al., 2019). Auditory attention often engages visual cortical areas (Cate et al., 2009), especially when the child “visualizes” the word as an association in an attempt to recall the word – i.e. in response to retaining the word “boat,” the child may visualize an image of the boat. Additionally, visual cortical areas in association with parietal association cortical regions are part of the dorsal stream network intimately involved in guided attention (Deco & Rolls, 2005).

We also found that hippocampal-thalamic connectivity was associated with CVLT. Thalamic projections have been shown to be particularly vulnerable to biomechanical, shear-strain deformation in TBI (Bian & Mao, 2020; Cosgrove et al., 2022; Dennis et al., 2021; Mofakham et al., 2022), where even in mild-TBI, diminished integrity of thalamic radiations occurs and relates to behavioral and cognitive outcome (Ware et al., 2020, 2022). The original Papez circuit delineates projections from hippocampus to mammillary bodies via the fornix, then to anterior thalamic nuclei (ATN) of the thalamus, and on to cingulate and posterior parietal cortex, and finally back to the hippocampus through parahippocampal cortex. The ATN are believed to be a part of an ‘extended hippocampal system’, which is involved in memory encoding (Aggleton et al., 2010; Sweeney-Reed et al., 2021). Lesions or degeneration of the ATN lead to impairments along this tract, impairing memory encoding (Sweeney-Reed et al., 2021). Thus, it is not surprising that reduced connectivity between the hippocampus and thalamus among TBI patients in our study, was associated with reduced memory performance.

Our structural connectivity findings are supported by our functional connectivity analysis, which similarly demonstrated a positive association between hippocampal network connectivity and memory performance. Similar to our structural connectivity analysis, the FC associations were found in the left hippocampal network. Consistent with prior studies, the hippocampal FC network in our analysis overlaps with the Default Mode Network, a set of brain regions that tend to be more active during “rest” and involved in, among other functions, episodic memory (Buckner et al., 2008; Raichle et al., 2001; Vincent et al., 2006). The TBI subjects with the highest CVLT-scores showed hippocampal functional connectivity that was similar to control subjects at comparable performance. This relationship with CVLT is consistent with prior studies of resting-state FC in children (Riggins et al., 2016) and older adults (Wang et al., 2010) that show a positive association of within-network hippocampal FC and memory performance.

### Importance of multimodality neuroimaging approach

2hile the vulnerability of the hippocampus to severe-TBI has been well established (Bigler et al., 2002; Dennis et al., 2021; Wilde et al., 2007), the current investigation is the first to take a multimodality neuroimaging analysis approach to examine hippocampal connectivity and network factors that most robustly relate to verbal memory in an adolescent severe-TBI sample. As mentioned in the introduction, merely assessing hippocampal volume or whether a lesion has been present within the hippocampal formation post-TBI has not necessarily predicted memory outcome. As demonstrated in the current investigation, the hippocampus, while a critical, central component to the memory network, happens to be just one component of the network. The participants in the current investigation, while having sustained a severe TBI had, nonetheless, sufficiently recovered to be able to cooperate with the cognitive assessment process and were between 1–2 years post injury. As such, the neuropsychological assessment process was undertaken at a point where sufficient plasticity, adaptation, accommodation and/or recovery of memory networks had occurred. What the current investigation demonstrates is that memory performance relates to the integrity of the entire memory network, not just a single component. Following injury, the hippocampus and related medial temporal lobe structures have some capacity for plasticity (Schumm et al., 2022) and there is also the potential for neurogenesis within the injured hippocampus (Rizk et al., 2021). How well hippocampal neurons may still participate in generating neural connectivity within the network may be key to how other systems and pathways in the memory network react to injury and potentially compensate to whatever hippocampal pathology may be present (M. Dennis et al., 2014; Sta Maria et al., 2019). Also, as shown in Fig. 2, even though this was a restricted sample of severe-pediatric-TBI, a number of the severe-TBI cases had hippocampal volumes that were entirely within the range of the TD control sample. Accordingly, interrogating the entire hippocampal network provides a more comprehensive view of injury-induced dysfunction and compensatory changes. From a practical perspective, however, hippocampal volume and/or fornix FA measures are the most straightforward to calculate on a clinical basis and appear to represent valid biomarkers of memory impairment in severe-TBI in children.

Some limitations must be considered when interpreting the results of our study. First, the relatively small sample size in our TBI cohort affects estimates of the quantitative neuroimaging measures in terms of variance and reliability, which in turns limits the generalizability of the findings. Also, site effects must be considered when interpreting results from any multi-site neuroimaging study. To minimize site-to-site variation, imaging protocols were standardized across sites. We also conducted an analysis of the NIST PVP diffusion phantom (Boss et al., 2015) scanned across 6 of the sites. The findings of that analyses (Guerrero-Gonzalez et al., 2022) did not reveal significant variations in diffusion measures across sites. Nonetheless, measurement variation across scanners and sites cannot be entirely ruled out as a contributing factor in this study. On the other hand, severe-TBI tends to produce significant changes in brain structure, manifested as generalized and regional volume loss (Beauchamp et al., 2011; Wilde et al., 2007), and MRI has been successfully used in other multi-site studies of adults (Palacios et al., 2022) and children (Bigler et al., 2013) with TBI. Normative control data and/or human traveling phantoms at each site could be employed in future studies to further address harmonization across sites but were not feasible here. Regardless, the group differences we observed in volumetric and DTI measures were robust and occurred in the expected direction, and the associations between hippocampal imaging findings and memory dysfunction we identified are biologically plausible.

## Conclusion

This analysis included hippocampal volume and fornix microstructure, as well as structural and functional hippocampal connectivity in relation to memory and learning. We found significant reductions in hippocampal volume in TBI subjects compared to controls, and in TBI subjects the hippocampal volume was associated with memory and learning performance on the CVLT. FA in the fornix of the TBI group significantly decreased with respect to the TD group. Also, in TBI reduced fornix FA correlated with CVLT performance. Further, we found statistically significant linear associations for CVLT and FBC of hippocampus to thalamus and calcarine sulcus. Functional connectivity analysis similarly demonstrated a positive association between left hippocampal network connectivity and memory performance. This multi-modal interrogation of the hippocampal network provides a more comprehensive look into injury-induced dysfunction and compensatory changes.

### Supplementary information

Below is the link to the electronic supplementary material.

Supplementary file1 (DOCX 14.2 MB)

## Data Availability

The participants of this study did not consent to public sharing of data. Thus, data cannot be published on public repositories. The authors are open to explore data sharing alternatives with proper procedures and documentation that comply with relevant review protocols.

## Code availability

All relevant functions and libraries that were used from existing packages (e.g. MRtrix3, TractSeg, etc.) are detailed in the pertinent sections of the manuscript or Supplement.
