# Supplementary material for: Multi-modal MRI of hippocampal morphometry and connectivity after pediatric severe TBI
Source: Brain Imaging Behav. 2023 Nov 13;18(1):159–70. doi: 10.1007/s11682-023-00818-x (PMC10844146; doi:10.1007/s11682-023-00818-x)
Supplement: Supplementary file 1 — Supplementary file1 (DOCX 14.2 MB) [file 11682_2023_818_MOESM1_ESM.docx]

**Supplement**

*Framework for Volumetrics, Microstructure, and Hippocampal Connectomes Derivation*

*Volumetrics Processing*

Initial quality assessment was done by visual inspection of the T1w images, and all were found acceptable for further analysis. Structural T1w images were processed using ‘*recon-all’* in Freesurfer (Fischl et al., 2002). Segmentations obtained were visually inspected and manual corrections performed when segmentation errors were encountered.

*Diffusion Processing*

Distortion, translation and rotation from bulk head motion and eddy currents were corrected using the eddy tool (Andersson & Sotiropoulos, 2016) in the FMRIB Software Library (FSL, version 6.0) with outlier replacement enabled (Andersson et al., 2016). The T1-weighed images were used to perform anatomically constrained tractography (ACT). In the cases of visually apparent atrophied tissue, we manually delineated those regions as binary masks in the ACT to avoid seeding from or tracking through those regions. Gradient directions were corrected for image rotations from image registration (Leemans & Jones, 2009). Since field maps were not available, EPI-related geometric distortions were corrected using an in-house image spatial normalization approach. This aligned the diffusion scan images to the structural T2- and T1-weighted images using both rigid body and diffeomorphic image co-registration, with the latter constrained along the phase encoding (PE) direction of the diffusion images. The rigid body stage was performed using boundary based registration with FreeSurfer’s ‘*bbregister*’ and the PE-constrained non-linear registration stage was done using ‘*antsRegistration*’ in ANTs (Avants et al., 2011). Ultimately, the diffusion data were up-sampled and aligned to the T1w image by applying the resulting transforms in a single step using ‘*antsApplyTransforms’* with BSpline interpolation. The rotation component of the rigid body transformation was applied to the diffusion gradient directions.

Diffusion tensors were estimated for each voxel using the robust estimation of tensors by outlier rejection (RESTORE) algorithm as part of the diffusion imaging in python (DIPY) software package (Garyfallidis et al., 2014). Fractional anisotropy maps were generated from the voxel-wise estimates of the diffusion tensor.

Additionally, white matter fiber Orientation Distribution Function (fODF) maps were estimated for use in subsequent steps including the segmentation of the fornix and in whole-brain fiber tracking (required for generating structural connectomes). This process was conducted using the MRtrix3 software package (Tournier et al., 2019) and started with estimation of tissue-specific response functions using ‘*dwi2response’* with the multi-tissue ‘*dhollander*’ algorithm (Dhollander et al., 2016). Individual response functions were then averaged across the TD group in order to estimate group mean response functions. At the individual level, Single-shell 3-Tissue CSD (constrained spherical deconvolution) (Dhollander & Connelly, 2016) was used to estimate tissue specific fODFs by deconvolving the signal with the three separate response functions. The estimation of CSF and gray matter fODFs is helpful in decontaminating the white matter fODF in voxels containing other tissue types like CSF and gray matter. Minimizing partial volume effects leads to more precise estimates of the white matter fODF and improved fiber tracking (Jeurissen et al., 2014), especially in structures like the fornix bordering the ventricles.

*Fornix FA*

Segmentation of the fornix bundles was carried out using TractSeg (Wasserthal et al., 2018), which uses a pre-trained convolutional neural network to create region-specific tractograms. In this process, the directions for the most dominant fiber populations at each voxel, known as peaks, were first extracted from the white matter fODF using the ‘*sh2peaks’* tool from MRTrix3 (Jeurissen et al., 2013; Tournier et al., 2019). These were then fed to TractSeg, which performed a multi-step process to derive the fornix streamline bundle. These set of steps included segmentation of regions that encompass the ﻿white matter tract as probability masks, definition of ‘start’ and ‘end’ regions for each mask, and segmentation of tract orientation (or peak) maps (TOMs) (Wasserthal et al., 2018). The process culminated with probabilistic fiber tracking within the TOMs using the segmented masks for seeding and using the start- and end- regions as inclusion points creating a bundle specific tractogram. Finally, FA was sampled using this segmentation of the fornix for each subject.

*Hippocampal Structural Connectome*

A hippocampus specific structural connectome was derived from the TD sample and applied to participants in the TBI group. In this process, SIFT2-filtered probabilistic whole-brain fiber tracking off the fODF maps (Smith et al., 2015; Tournier et al., 2019) was combined with 164 cortical and subcortical gray matter regions to produce individual structural connectomes (Figure S3). FreeSurfer was used in estimating the gray matter regions based on the Destrieux atlas (Destrieux et al., 2010; Fischl et al., 2002). These regions were used as nodes for estimating pairwise connectivity. The measure of connectivity was chosen to be the fiber bundle capacity (FBC), which represents the ability of a white matter pathway to carry information (Smith et al., 2020). FBC is computed from the sum of the streamline weights that form a fiber bundle, where weights represent the contribution of each streamline to the fiber density at each voxel estimated from the fODFs and are output as part of the SIFT2 filtering process (Smith et al., 2020).

In deriving the hippocampus-specific connectome, the full-brain connectomes were first averaged across participants in the TD group. Then, hippocampus specific connectivity was extracted from the mean connectome representing only those connections to or from the hippocampus. The resulting connectome was then sorted in descending order with respect to connectivity values (Figure S4). Note how the curve in this plot decays fast and begins to level off around this 5% threshold. To the right of this ‘elbow’ on the curve the remaining regions have similar connectivity, with lower values (tending to 0) than those to the left of the ‘elbow’. The top 5% most highly connected regions are therefore selected to define the hippocampus connectome.

*Functional MRI preprocessing*

All functional MRI (fMRI) data processing was performed using the AFNI software package. Resting-state fMRI echo-planar imaging (EPI) timeseries data were processed by removing the first 3 volumes to allow the magnetization to reach steady-state, performing rigid-body volume realignment, and performing slice-timing correction. Time points where the volume-to-volume motion (computed as the Euclidean norm of the temporal difference of the 6 realignment parameters) exceeded 0.2mm were excluded from further analysis. The fMRI time series data were then bias-field corrected and aligned to the T1-weighted structural image using 12-parameter affine transformations. Each subject’s T1-weighted image was non-linearly aligned to the MNI template brain. The combined EPI-to-T1 and T1-to-template transformations were concatenated and applied as a single interpolation to warp the original preprocessed fMRI data into template space. The T1w image was segmented into gray matter, white matter, and CSF using the tool ‘*fast’* from FSL. The preprocessed fMRI signal averaged over eroded white matter and CSF masks, the 6 realignment parameters, and the temporal derivatives of these signals were then regressed out of the data using multiple linear regression. Finally, the data were smoothed by an iterative procedure (AFNI’s 3dBlurToFWHM) to achieve a final spatial smoothness of 8mm.

*Hippocampus functional connectivity*

A mask of left and right hippocampus was defined using the Talairach Daemon. Functional connectivity of each hippocampus was computed by first averaging the pre-processing fMRI data over each of these masks, and then computing the temporal Pearson’s correlation with all other voxel time series. The network of brain regions significantly connected to each hippocampus (“hippocampal network”) was determined by computing a 1-sample t-test of the Fisher-Z transformed voxel-wise connectivity maps in the control subjects. Voxels where the group-level functional connectivity exceeded a Bonferroni corrected p-value of 0.05 (i.e. an individual voxel threshold of 0.05 divided by the number of voxels in the brain, 233934) were considered to be significantly connected. Figure S7 shows the hippocampal network combined for both left and right hippocampus seeds in control subjects.

**
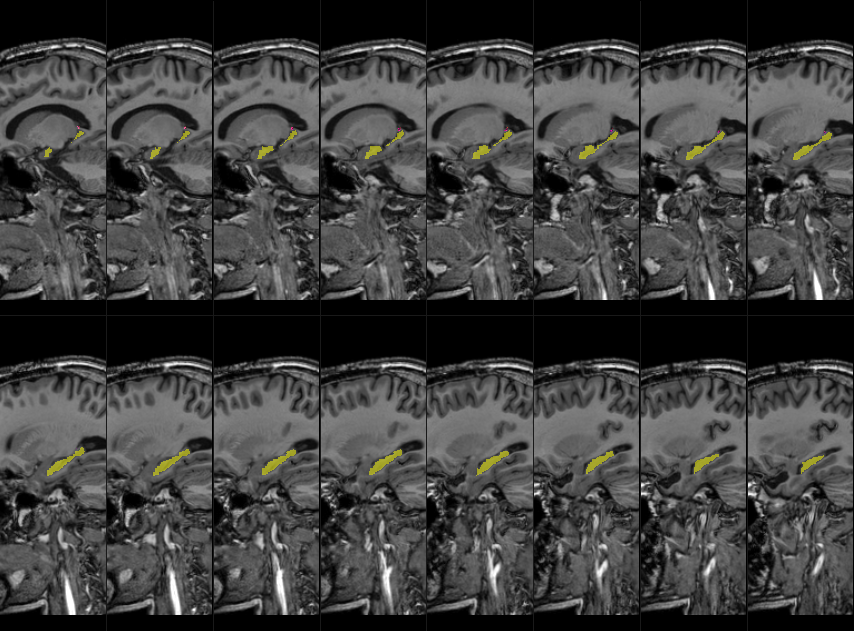
**

Figure S1. Representative hippocampus segmentation. Segmentation depicted in yellow is from one of the TBI subjects. Sagittal slices were selected to cover head, body, and tail of hippocampus.

**
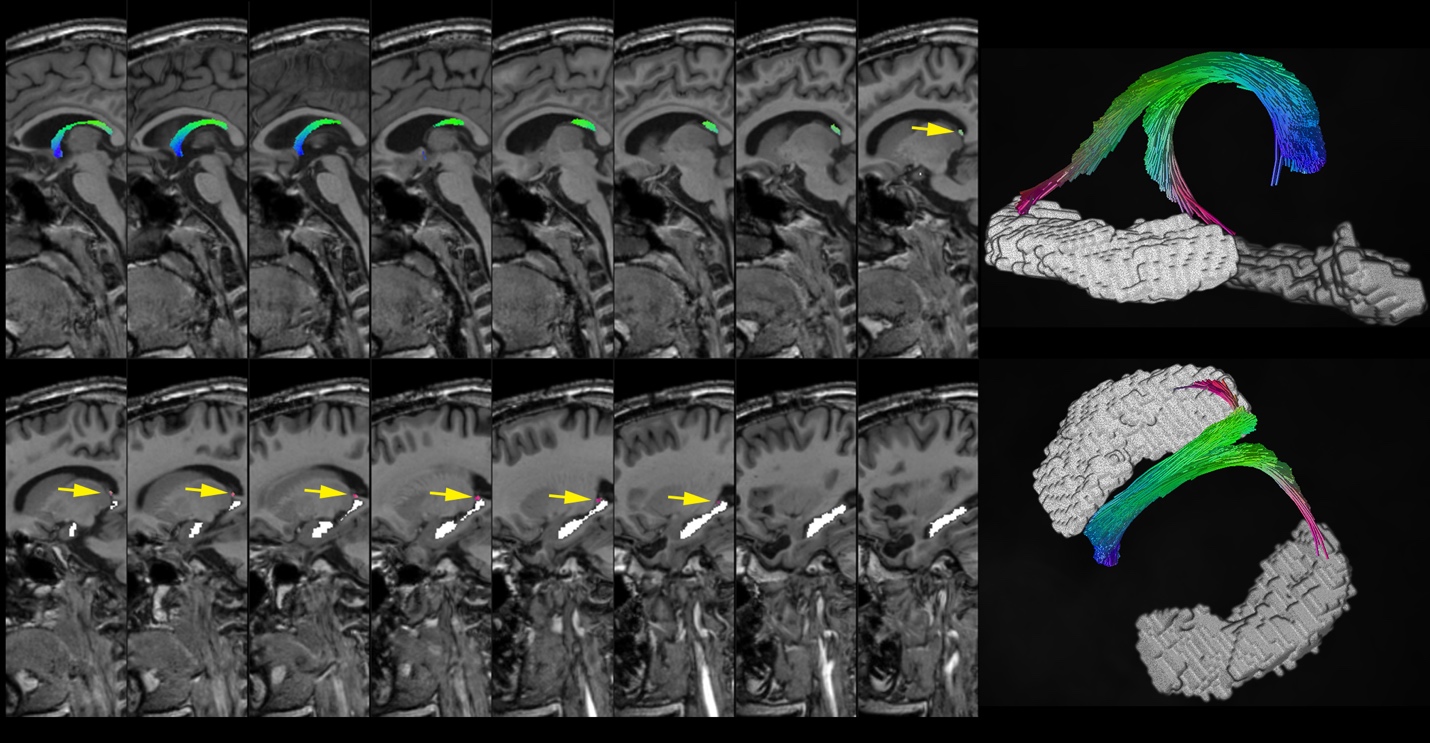
**

Figure S2. Representative fornix segmentation. Streamlines segmentation directionally encoded (red:right-left, green:anterior-posterior; blue:inferior-superior) from one of the TBI subjects. Sagittal slices were selected to cover fornix from mid-sagittal plane to the hippocampus. Hippocampus segmentation is shown in white. Yellow arrows point to the thin sections of the fornix as it approaches and eventually joins the hippocampus. 3-D renderings of the fornix riding on the hippocampus are also shown for the same subject.


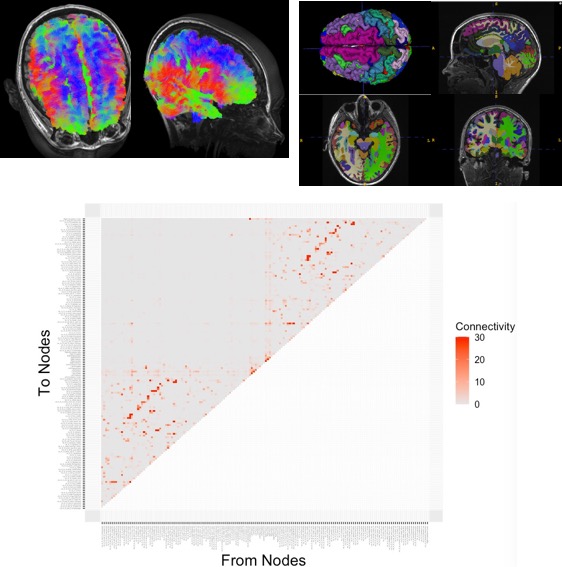


Figure S3. The Destrieux structural connectome. Whole brain probabilistic fiber tracking is combined with 164 cortical and subcortical gray matter labels derived from the Destrieux parcellation in FreeSurfer, to produce a connectivity matrix where each element represents the connectivity between each pair of nodes.


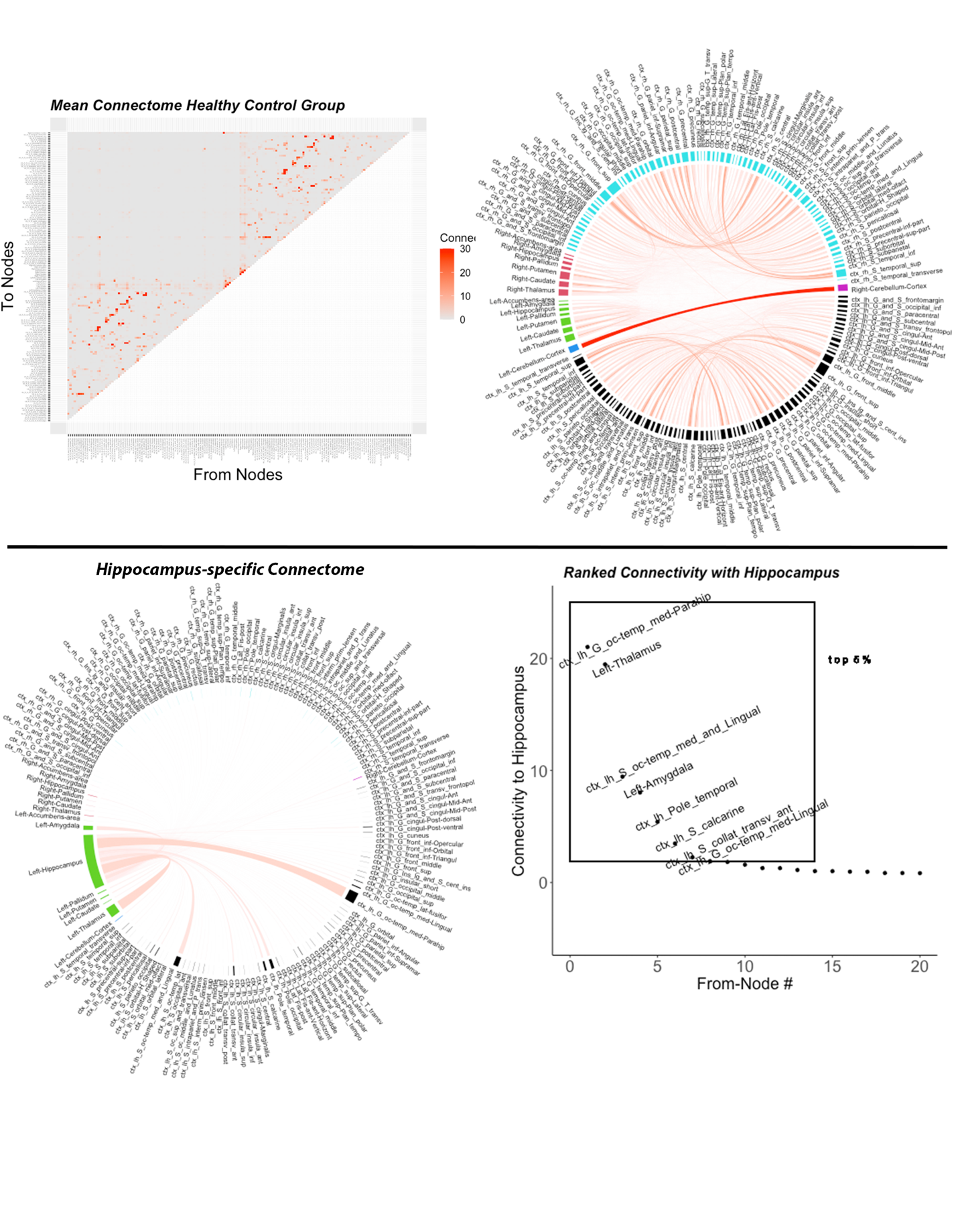


Figure S4. Deriving the hippocampal structural connectome. Top panel: Mean connectome across the control group represented as a connectivity matrix (left) and a connectogram (right). Lower panel: Hippocampus specific connectogram (left) and connected regions ranked from highest to lowest connectivity. The top 5% most highly connected regions are selected to define the hippocampus connectome.


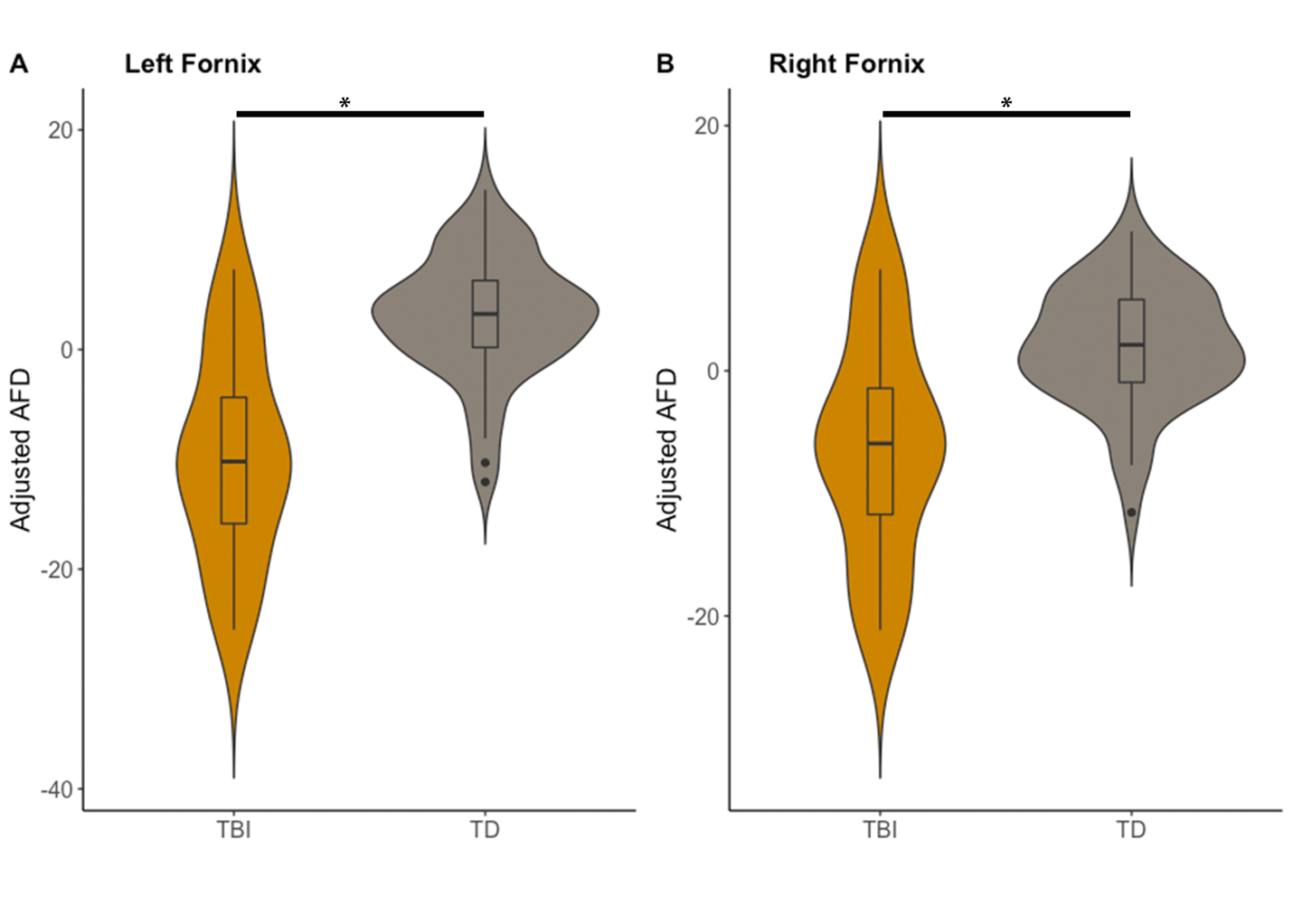


Figure S5. Group comparison of fornix apparent fiber density (AFD). A-B, group comparison of left and right fornix AFD between TBI (yellow) and control cohort (gray), adjusted for age and sex; *p<0.05.


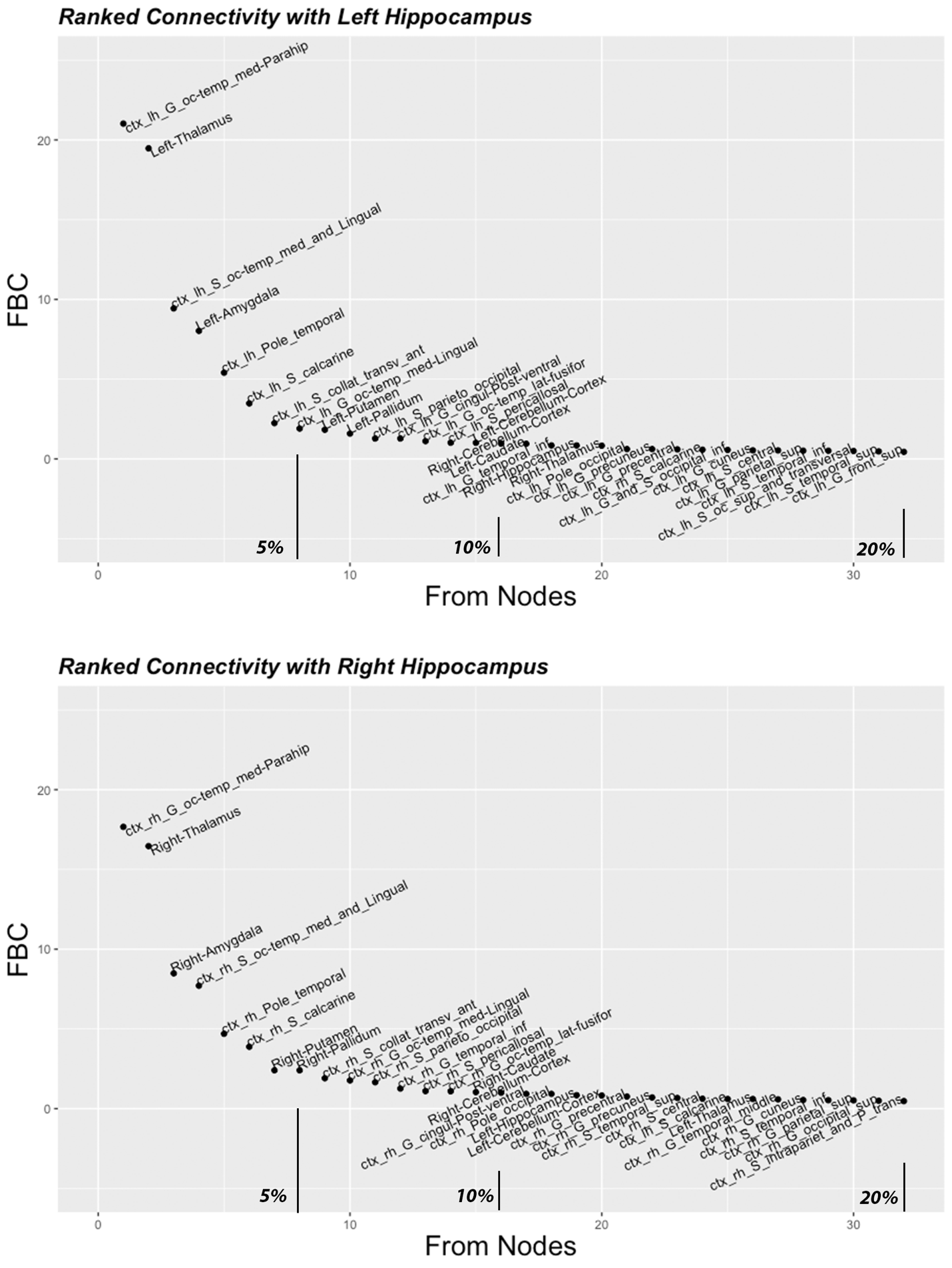


Figure S6. Ranked hippocampal connectivity. Regions were ranked from higher to lower FBC. Plots show top 5, 10, and 20% most connected regions.

Figure S7. Functional connectivity of the hippocampus. Network of brain regions significantly connected to the left (A) and right (B) hippocampus in control subjects (p<0.05, corrected).

**References**

Andersson, J. L. R., Graham, M. S., Zsoldos, E., & Sotiropoulos, S. N. (2016). Incorporating outlier detection and replacement into a non-parametric framework for movement and distortion correction of diffusion MR images. *NeuroImage*, *141*, 556–572. https://doi.org/10.1016/j.neuroimage.2016.06.058

Andersson, J. L. R., & Sotiropoulos, S. N. (2016). An integrated approach to correction for off-resonance effects and subject movement in diffusion MR imaging. *NeuroImage*, *125*, 1063–1078. https://doi.org/10.1016/j.neuroimage.2015.10.019

Avants, B. B., Tustison, N. J., Song, G., Cook, P. A., Klein, A., & Gee, J. C. (2011). A reproducible evaluation of ANTs similarity metric performance in brain image registration. *NeuroImage*, *54*(3), 2033–2044. https://doi.org/10.1016/j.neuroimage.2010.09.025

Destrieux, C., Fischl, B., Dale, A., & Halgren, E. (2010). Automatic parcellation of human cortical gyri and sulci using standard anatomical nomenclature. *NeuroImage*, *53*(1), 1–15. https://doi.org/10.1016/j.neuroimage.2010.06.010

Dhollander, T., & Connelly, A. (2016). A novel iterative approach to reap the benefits of multi-tissue CSD from just single-shell (+ b= 0) diffusion MRI data. *Proc ISMRM 2016*, *24*, 3010.

Dhollander, T., Raffelt, D., & Connelly, A. (2016). *Unsupervised 3-tissue response function estimation from single-shell or multi-shell diffusion MR data without a co-registered T1 image*. ISMRM Workshop on Breaking the Barriers of Diffusion MRI, Lisbon, Portugal.

Fischl, B., Salat, D. H., Busa, E., Albert, M., Dieterich, M., Haselgrove, C., van der Kouwe, A., Killiany, R., Kennedy, D., Klaveness, S., Montillo, A., Makris, N., Rosen, B., & Dale, A. M. (2002). Whole Brain Segmentation. *Neuron*, *33*(3), 341–355. https://doi.org/10.1016/S0896-6273(02)00569-X

Garyfallidis, E., Brett, M., Amirbekian, B., Rokem, A., van der Walt, S., Descoteaux, M., Nimmo-Smith, I., & Dipy Contributors. (2014). Dipy, a library for the analysis of diffusion MRI data. *Frontiers in Neuroinformatics*, *8*. https://doi.org/10.3389/fninf.2014.00008

Jeurissen, B., Leemans, A., Tournier, J.-D., Jones, D. K., & Sijbers, J. (2013). Investigating the prevalence of complex fiber configurations in white matter tissue with diffusion magnetic resonance imaging: Prevalence of Multifiber Voxels in WM. *Human Brain Mapping*, *34*(11), 2747–2766. https://doi.org/10.1002/hbm.22099

Jeurissen, B., Tournier, J.-D., Dhollander, T., Connelly, A., & Sijbers, J. (2014). Multi-tissue constrained spherical deconvolution for improved analysis of multi-shell diffusion MRI data. *NeuroImage*, *103*, 411–426. https://doi.org/10.1016/j.neuroimage.2014.07.061

Leemans, A., & Jones, D. K. (2009). The *B* -matrix must be rotated when correcting for subject motion in DTI data. *Magnetic Resonance in Medicine*, *61*(6), 1336–1349. https://doi.org/10.1002/mrm.21890

Smith, R. E., Tournier, J.-D., Calamante, F., & Connelly, A. (2015). SIFT2: Enabling dense quantitative assessment of brain white matter connectivity using streamlines tractography. *NeuroImage*, *119*, 338–351. https://doi.org/10.1016/j.neuroimage.2015.06.092

Smith, R., Raffelt, D., Tournier, J.-D., & Connelly, A. (2020). *Quantitative streamlines tractography: Methods and inter-subject normalisation* [Preprint]. Open Science Framework. https://doi.org/10.31219/osf.io/c67kn

Tournier, J.-D., Smith, R., Raffelt, D., Tabbara, R., Dhollander, T., Pietsch, M., Christiaens, D., Jeurissen, B., Yeh, C.-H., & Connelly, A. (2019). MRtrix3: A fast, flexible and open software framework for medical image processing and visualisation. *NeuroImage*, *202*, 116137. https://doi.org/10.1016/j.neuroimage.2019.116137

Wasserthal, J., Neher, P., & Maier-Hein, K. H. (2018). TractSeg—Fast and accurate white matter tract segmentation. *NeuroImage*, *183*, 239–253. https://doi.org/10.1016/j.neuroimage.2018.07.070
